# Identification of a major QTL conferring resistance to *wheat yellow mosaic virus* derived from the winter wheat ‘Hokkai 240’ on chromosome 2AS

**DOI:** 10.1270/jsbbs.23079

**Published:** 2024-07-18

**Authors:** Kenji Kawaguchi, Takehiro Ohki, Goro Ishikawa, Mitsuru Sayama, Yohei Terasawa, Shunsuke Oda, Masaya Fujita, Miwako Ito, Koichi Hatta

**Affiliations:** 1 NARO Hokkaido Agricultural Research Center, Shinsei, Memuro, Hokkaido 082-0081, Japan; 2 Department of Agro-Environmental Science, Obihiro University of Agriculture and Veterinary Medicine, Obihiro, Hokkaido 080-8555, Japan; 4 NARO Hokkaido Agricultural Research Center, Hitsujigaoka, Toyohira, Sapporo, Hokkaido 062-8555, Japan; 5 NARO Institute of Crop Science, Tsukuba, Ibaraki 305-8518, Japan; 7 NARO Kyushu Okinawa Agricultural Research Center, 496 Izumi, Chikugo, Fukuoka 833-0041, Japan

**Keywords:** *Triticum aestivum*, *wheat yellow mosaic virus* (WYMV), disease resistance, DNA marker

## Abstract

Wheat yellow mosaic disease is a soilborne disease caused by *wheat yellow mosaic virus* (WYMV). Symptoms include yellow mosaic coloring of leaves, stunting, and growth inhibition. Here we conducted a detailed analysis of resistance to this virus in winter wheat ‘Hokkai 240’ by carrying out inoculation tests of WYMV and conducting field tests. The resistance level observed in ‘Hokkai 240’ was compared with those in varieties harboring known resistance genes. In the inoculation tests, ‘Hokkai 240’ showed resistance to WYMV Pathotypes I and II and partial resistance to Pathotype III. This result was contrary to the sensitive responses to the three pathotypes exhibited by the variety harboring resistance gene on chromosome 2DL. In fields infected with WYMV Pathotypes II and III, ‘Hokkai 240’ plants exhibited few disease symptoms and little proliferation of the virus. By analyzing the quantitative trait loci (QTLs) in recombinant inbred lines from a cross between ‘Hokkai 240’ and ‘Nanbukomugi’, a single major QTL, *Q.Ymhk*, from ‘Hokkai 240’, which had significant effects on Pathotypes II and III of the virus, was detected in the proximity of *snp4212* and *snp4215* mapped on chromosome 2AS. These results indicate that *Q.Ymhk* may be useful for developing broad resistance to WYMV in wheat breeding programs.

## Introduction

Wheat yellow mosaic disease is soilborne and causes serious damage to wheat crops in Japan and China (Han *et al.* 2000, Inoue 1969, Sawada 1927). *Wheat yellow mosaic virus* (WYMV) from the genus *Bymovirus* in the family *Potyviridae* is the causal agent of the disease and is transmitted by *Polymyxa graminis*, a eukaryotic obligate biotrophic parasite of plant roots (Inoue 1969, Ohto 2005). WYMV infection triggers leaf yellowing, the accumulation of anthocyanins in leaves, stunting, and dwarfing symptoms (Ohto 2005). In severe cases, the virus negatively affects grain weight and/or ear numbers (Ohto 2005). Controlling WYMV by disinfecting the soil is expensive, and crop rotation is insufficient to reduce the levels of the virus, due to its ability to survive long-term in dormant *P. graminis* spores that reside in the soil (Ohto 2005). Therefore, it is necessary to breed wheat varieties that are resistant to WYMV.

In Japan, WYMV was first reported on Kyushu Island in the early part of the 20th century (Sawada 1927). By the end of the 1990s, it had been transmitted to the northern island (Hokkaido), which is the major wheat-producing area in Japan (Kusume *et al.* 1997), and then spread throughout the main wheat-growing regions (Horita *et al.* 2011). To address this situation, breeders have made efforts to develop varieties resistant to WYMV (Suzuki *et al.* 2022). Isolates of WYMV in Japan are classified into three pathotypes (I, II, and III) based on variations in the viral RNA sequence and differences in their abilities to infect wheat varieties (Ohki *et al.* 2014, Ohto *et al.* 2006). Pathotype I is found mainly in western and central Japan (Kinki, Chugoku, Shikoku, Kyushu, Chubu, and Kanto regions), Pathotype II in northern Japan (Tohoku and Hokkaido regions), and Pathotype III in part of the Kyushu region (Ohki *et al.* 2014, Ohto *et al.* 2006). The three pathotypes can be distinguished based on the responses of three varieties ‘Nanbukomugi’, ‘Fukuhokomugi’, and ‘Hokkai 240’ following mechanical inoculation of the leaves. Pathotype I infects ‘Nanbukomugi’ and ‘Fukuhokomugi’, Pathotype II infects only ‘Nanbukomugi’, and Pathotype III infects all three varieties (Ohto *et al.* 2006).

To date, many genetic studies have been conducted on WYMV resistance genes, and molecular markers have been developed for marker-assisted selection of wheat lines with resistance to the disease. Among previously reported genes and quantitative trait loci (QTLs), those on chromosomes 2DL, 3BS, 5AL, and 6DS are considered to contribute most to WYMV resistance. In the Chinese variety ‘Xifeng’ (syn. ‘Nishikazekomugi’), a QTL on chromosome 5AL is associated with partial resistance to Pathotype I (Kojima *et al.* 2019). *Qym4* on chromosome 6DS was found to confer resistance to Pathotype II in the Japanese variety ‘OW104’ (Yamashita *et al.* 2020). In the US variety ‘Madsen’, two QTLs for resistance to Pathotype II, *Qym1* (*YmMD*) and *Qym2*, were identified on chromosomes 2DL and 3BS, respectively (Kojima *et al.* 2019, Suzuki *et al.* 2015, Takeuchi *et al.* 2010). Genes and QTL in different germplasms have been detected at similar locations on chromosome 2DL: *YmYF* from a Chinese variety ‘Yangfu 9311’ (Liu *et al.* 2005a); *YmIb* from a European variety ‘Ibis’ (Nishio *et al.* 2010); and *Q.Ymym* from a Japanese variety ‘Yumechikara’ (Kojima *et al.* 2015). It remains to be confirmed whether these QTLs are the same locus, but varieties with a resistance allele in this region show resistance to all three pathotypes of the virus (Kojima *et al.* 2019). Recently, a causative gene *Ym2* at *the* *Qym2* on chromosome 3BS was identified, and it encodes a coiled-coil nucleotide-binding site leucine-rich repeat protein (Mishina *et al.* 2023). Although the understanding of genetic control for WYMV resistance is advancing, further analysis to explore resistant genes should be required, as the number of reliable WYMV resistance genes is limited.

‘Hokkai 240’ has been reported as one of the useful germplasms for the breeding of WYMV resistance in Japan, as it shows resistance to Pathotypes I and II in inoculation and field tests (Oda and Kashiwazaki 1989, Ohto *et al.* 2006). However, reaction to Pathotype III in the field and genetic basis for resistance are unknown. It would, therefore, be useful to determine the details of resistance in ‘Hokkai 240’ and identify the genetic factors that could contribute to breeding WYMV-resistant varieties.

In this study, we conducted phenotypic and genetic analyzes of WYMV resistance in ‘Hokkai 240’. First, we evaluated the resistance of ‘Hokkai 240’ by performing field and inoculation tests with the pathotype III of WYMV. In its experiments, Hokkai 240 showed consistent level of resistance in the field and inoculation tests. Furthermore, QTL analysis was conducted to investigate the resistance to Pathotypes II and III following a field test using recombinant inbred lines (RILs) from a cross between ‘Hokkai 240’ and ‘Nanbukomugi’. The findings indicated that resistance in ‘Hokkai 240’ to Pathotypes II and III is controlled by a single major QTL on chromosome 2AS.

## Materials and Methods

### Plant materials

The test materials and details about their levels of resistance are listed in Supplemental Table 1. Field tests of Pathotype II and III were performed using ‘Hokkai 240’ and ‘Nanbukomugi’. RILs from a cross between ‘Hokkai 240’ and ‘Nanbukomugi’ were generated using the single-seed descent method. The reactions to Pathotypes I, II, and III of WYMV were tested by mechanical inoculation of the wheat varieties ‘Nanbukomugi’, ‘Fukuhokomugi’, ‘Hokkai240’, and ‘Yumechikara’. ‘Yumechikara’ exhibits resistance to the three pathotypes of WYMV in field tests (Kobayashi *et al.* 2020, Kojima *et al.* 2015, Tabiki *et al.* 2011), but its response to WYMV inoculation is unknown.

To identify the genotype at loci associated with WYMV resistance in major winter wheat varieties currently cultivated in Hokkaido, we used ‘Yumechikara’, ‘Kitahonami’, ‘Kitanokaori’, and ‘Tsurukichi’.

### Evaluation of disease severity in field tests with WYMV Pathotype III

To investigate resistance to Pathotype III, seeds of ‘Hokkai 240’ and ‘Nanbukomugi’ were sown in early November in paddy backcrop field in Yanagawa City, Fukuoka Prefecture, Kyushu region (33.1°N, 130.4°E) in 2005, 2006, and 2007. This field was reported to be infested with Pathotype III (Kojima *et al.* 2019, Ohki *et al.* 2014). In 2006 and 2007, the RILs (F_6–7_) population was examined. One plot was sown per line, and it contained 10–20 seeds per 0.2 m row. Rows were 0.2 m apart. Disease severity was evaluated by visual observation according to the disease index (DI). The DI was based on a seven-point scale with minor modifications described by Nishio *et al.* (2010) as follows: 0, no disease symptoms; 1, slight yellowing of leaves; 2, characteristic symptoms (e.g., distinct yellowing, anthocyanins accumulation, crusty streaks) observed in some plants; 3, symptoms widespread in all plants; 4, severe symptoms and growth inhibition observed in all plants; 5, some individuals in the plot had died; and 6, all individuals in the plot had died. The plots were surveyed three times during February and March, and the highest DI value the survey period was determined as the representative value for the plot. Because of poor germination in 2006 and 2007, DI could not be assessed for some lines. Broad-sense heritability (*H^2^*) in RILs was calculated as the ratio of the genetic variance (*V_G_*) to the sum of environmental variance (*V_E_*) and *V_G_*. *V_E_* was the mean of the phenotypic variance of the parents; *V_G_* was calculated by subtracting *V_E_* from the phenotypic variance of the RILs.

### Reevaluation of resistance to WYMV in ‘Hokkai 240’ using inoculation tests

To reevaluate the response of ‘Hokkai 240’ to each Pathotype of WYMV, isolates from Tsukuba (Pathotype I), Date (Pathotype II), and Yanagawa (Pathotype III) (Ohki *et al.* 2014) were inoculated onto ‘Hokkai 240’, ‘Fukuhokomugi’, ‘Nanbukomugi’ and ‘Yumechikara’ at the 2.5–3-leaf stage. The isolates were mechanically inoculated by rubbing the leaves with carborundum and inoculum, which was prepared by grinding diseased leaves in a 10-fold volume of 50 mM phosphate buffer (pH 7.0). The inoculated plants were placed in a growth chamber (LPH-350SP, Nippon Medical & Chemical Instruments) under 14 h light/10 h dark conditions at 10°C for Pathotype I and II isolates or at 8°C for Pathotype III isolate. After 6 and 8 weeks, the second expanded leaf from the top (an uninoculated leaf) of each plant was sampled and examined for WYMV infection using double antibody sandwich enzyme-linked immuno-sorbent assay (DAS-ELISA) (Netsu *et al.* 2011). Leaves were ground in a 20-fold volume of phosphate-buffered saline with Tween-20 using a multi-bead shocker (Yasui Kikai) and analyzed using ELISA. The absorbance value at 405 nm was measured 2 h after adding the substrate, and samples showing more than twice the value of negative control were judged to be positive. The negative control was leaves of uninoculated plants grown in sterile soil.

### Evaluation of viral infection in field tests with WYMV Pathotype II

To investigate resistance to Pathotype II, trials were conducted in fields test in Hokkaido. Fields in Date (42.3°N, 140.5°E) and Obihiro (42.4°N, 143.1°E) were used in the 2017 and 2018 trials, respectively. Field in Date was reported to be infested with Pathotype II (Kojima *et al.* 2019, Ohki *et al.* 2014) and our preliminary test revealed that the field in Obihiro also infested with Pathotype II (data not shown). Plant materials were sown in early October. Each plot was sown with 10–20 seeds per 0.75 m row, and the rows were 0.3 m apart. Eight replicates of the RILs parental lines were grown in 2017 and six replicates were grown in 2018. Four replicates of the RILs (F_10–11_) were grown in 2017 and three were grown in 2018.

In Hokkaido, cold damage and snow mold occur during winter, making the evaluation of disease symptoms caused by WYMV. ‘Nanbukomugi’ was bred in other region (Laboratory of Wheat and Barley Breeding 1970) and might not be suitable for the winter environment of Hokkaido. Therefore, the degree of infection was evaluated by ELISA and not by visual observation. Leaves were sampled in early April after sowing the previous year and tested using ELISA. In each plot, one leaf from each of three randomly selected individuals was sampled, and tested using ELISA. ELISA methods were the same as for the inoculation test. Infection of lines with plots were determined based on the same criteria as that of inoculation test performed in 2017 trials. In the 2018 trials, the absorbance value of the test samples was generally high, so more than four times the value of negative control were considered to be infected. Infection rates of each line were recorded for QTL analysis. For example, if 1 out of 4 replicates was positive, the line was scored as 25% (1/4). Five lines were excluded from the QTL analysis, because the plants in these some test plots died from cold damage or snow mold in 2017. In addition, qualitative evaluation was used for verification of effectiveness of the marker that distinguish the resistant genotype. The lines were considered resistant if it was not infected in all plots and susceptible if infected in even one plot.

### DNA extraction

Approximately 50 mg of young leaf material were collected from each line, and the total genomic DNA was extracted in 300 μl extraction buffer containing 1 M KCl, 100 mM Tris-HCl (pH 8.0), and 10 mM ethylenediamine tetraacetic acid (EDTA; pH 8.0). DNA was precipitated with 100 μl isopropanol, washed with 200 μl 70% ethanol, and dissolved in 100 μl sterilized distilled water. Extracted DNA was used directly for the following analysis.

### Development of single-nucleotide polymorphism markers

A total of 480 single-nucleotide polymorphism (SNP) markers that showed polymorphism between the parental lines of the RILs and were distributed across the genomes were selected from previously developed amplicon sequencing markers (Ishikawa *et al.* 2020). The primers for the selected markers were mixed in a single tube, and genotyping via amplicon sequencing was performed following the protocol described by Ishikawa *et al.* (2018), except for the use of Platinum Multiplex PCR Master Mix (Thermo Fisher Scientific) instead of Multiplex PCR Master Mix (Qiagen). Markers with more than 15% missing data or showing skewed segregation were removed from further analysis.

### Mapping the flanking markers of genes associated with WYMV resistance

The parents of the RILs were genotyped to confirm that they possessed genes previously reported to be associated with WYMV resistance (Supplemental Table 2). PCR was performed in a total volume of 20 μl with 0.4 μM primer, Gotaq Master Mix (Promega), and 100 ng template DNA, using a T100 Thermal Cycler (Bio-Rad). PCR products were applied to a 3% agarose gel stained with Midori Green (Nippon genetics), separated by electrophoresis with TAE buffer, and the DNA bands were observed.

### Linkage map construction and QTL analysis

A linkage map of ‘Nanbukomugi’/‘Hokkai 240’ RILs was constructed using Joinmap^®^4.1 software (Van Ooijen 2006). Map distances were computed with the Maximum Likelihood mapping function with default settings. All markers were assigned to chromosomes based on the physical map from reference genome sequence (RefSeq) v2.1 of variety ‘Chinse spring’ (Zhu *et al.* 2021). Multiple QTL model (MQM) mapping was used for QTL analysis according to the resistant or susceptible in each line, and the DI by MapQTL^®^6 (Van Ooijen 2009). QTL analysis was carried out as described by Okano *et al.* (2020). First, interval mapping was performed, and the putative QTLs with LOD scores >3.0 were identified. Next, one marker was selected as a cofactor that showed the highest LOD peak at each putative QTL detected by interval mapping, and then MQM mapping was performed. If a new set of cofactors was selected based on the results of the first round of MQM mapping, MQM mapping was then repeated. The final set of cofactors was determined after multiple rounds of analysis. The 1000 replicate permutation test was conducted in MapQTL^®^6 to determine the genome-wide LOD threshold at the 1% level for each trait (Churchill and Doerge 1994).

We performed a Kompetitive allele specific PCR (KASP) assay using the primers (FAM: 5ʹ-gaaggtgaccaagttcatgctATGGTCATCCCAATATGCGC-3ʹ, HEX: 5ʹ-gaaggtcggagtcaacggattAATGGTCATCCCAATATGCGT-3ʹ, COM: 5ʹ-TAATGTTTAGCTTTTCTAATACAATGATAG-3ʹ), which were designed from the amplicon sequencing marker *snp4212* (Ishikawa *et al.* 2020). The assay was conducted in a 5-μL reaction volume containing KASP master mix (2.5 μL), KASP primer assay mix (0.07 μL), and 2 μL of DNA template (10 ng/μL). The cycling condition was as follows: 94°C for 15 min, 10 cycles of 94°C for 20 s, and 61°C–55°C in decrements of 0.6°C per cycle for 1 min, followed by 32 cycles of 94°C for 20 s and 55°C for 1 min. Fluorescent endpoints were read using the CFX384 Touch Real-Time PCR Detection System (Bio-Rad) and genotypes were called using CFX Manager 3.1 software in the allele discrimination mode.

## Results

### Disease severity in genetic resources during field tests against Pathotype III of WYMV

**Fig. 1.**
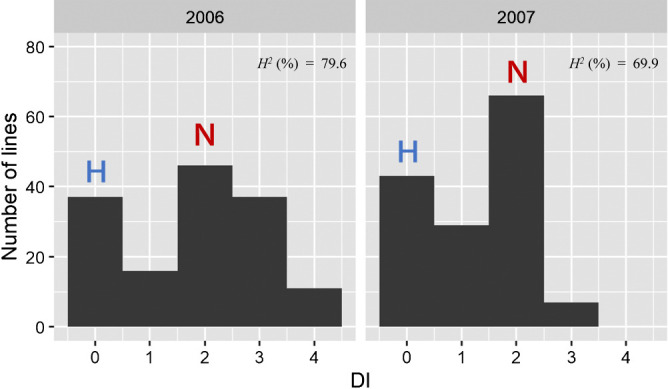
Distribution of disease index (DI) in ‘Nanbukomugi’/‘Hokkai 240’ Recombinant inbred lines (RILs) in the Pathotype III field test. Letters indicate the DI means of the parent (H = Hokkai 240, N = Nanbukomugi). *H^2^* means broad-sense heritability.

In the field tests against WYMV Pathotype III, ‘Hokkai 240’ had a lower DI, over three years (2005–2007) than ‘Nanbukomugi’ (Table 1). To examine whether the resistance of ‘Hokkai 240’ is a genetic trait, the DI of the RILs population was evaluated over 2 years in field tests (Fig. 1, Supplemental Table 3). The bimodal distributions of DI peaked at 0 and 2 during 2006 and 2007, respectively, and the Pearson correlation coefficient for the 2-year DI was *r* = 0.51 (*P* < 0.001). In addition, *H^2^* was 79.6% in 2006 and 69.9% in 2007. These results indicated the presence of genetic factor(s) controlling WYMV resistance in ‘Hokkai 240’.

### Differences in reactions to WYMV Pathotype III following mechanical inoculation

Hokkai 240 displayed resistance to Pathotype III in the field. Thereafter, inoculation tests were conducted to reevaluate the response to Pathotype III in the aboveground parts (shoots). The wheat varieties tested showed similar responses to Pathotypes I and II as those reported by Ohto *et al.* (2006) (Table 2). That is, ‘Nanbukomugi’ was infected by both pathotypes, ‘Fukuhokomugi’ was resistant to Pathotype II, and ‘Hokkai 240’ was resistant to both pathotypes. Following inoculation with Pathotype III, most ‘Nanbukomugi’ (22/23) and ‘Fukuhokomugi’ (18/23) plants succumbed to infection, but fewer ‘Hokkai 240’ (8/24) plants were infected. ‘Yumechikara’, which showed resistance to the three pathotypes in the field, was infected with three pathotypes at 6 weeks after inoculation. The analysis was terminated, however, because most ‘Yumechikara’ individuals at 8 weeks after inoculation showed clear disease symptoms.

### Resistance to Pathotype II in ‘Nanbukomugi’ × ‘Hokkai 240’ RILs

**Fig. 2.**
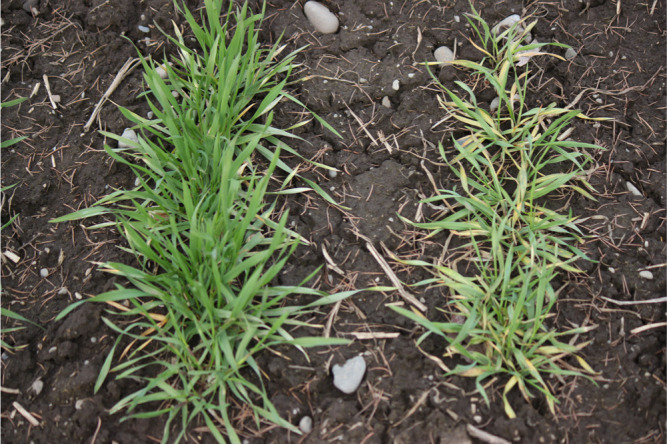
Appearance of ‘Hokkai 240’ (left) and ‘Nanbukomugi’ (right) in the field test of Pathotype II at the time of sampling for ELISA.

We conducted a further analysis of ‘Hokkai 240’ in the field at Hokkaido, where Pathotype II of WYMV was prevalent. ‘Nanbukomugi’ was infected, but ‘Hokkai 240’ was not infected over two years (Fig. 2, Table 3). The RILs had almost half of the lines infected in both years (Supplemental Table 4). In 2017, 68 of 146 lines were infected; in 2018, 69 of 151 lines were infected.

### QTL mapping of the WYMV resistance gene

**Fig. 3.**
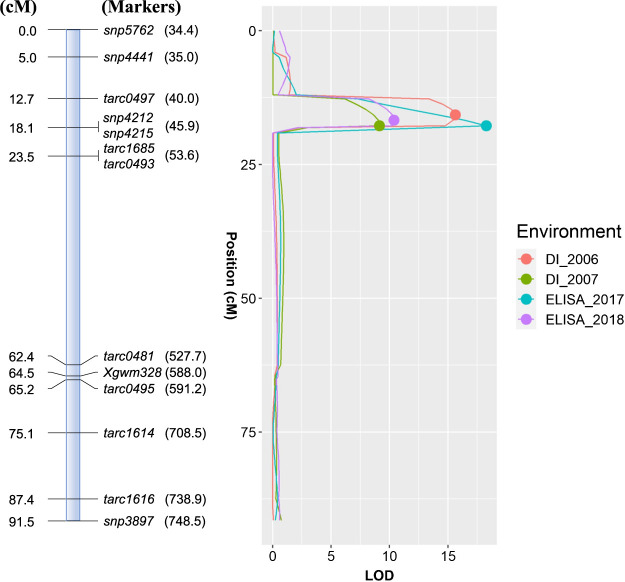
QTL analysis of chromosome 2A in ‘Nanbukomugi’/‘Hokkai 240’ RILs. The circles indicate the positions showing the maximum LOD score. The number in parentheses next to the marker name indicates the physical location of the ‘Chinese spring’ v2.1 (Zhu *et al.* 2021).

In the 151 RILs (F_10_) population derived from ‘Nanbukomugi’/‘Hokkai 240’, 21 linkage groups were constructed with a total genetic distance of 4600.0 cM, consisting of 334 SNPs and five flanking markers of QTLs associated with resistance to WYMV. The five flanking markers of the polymorphism were linked to previously reported genes or QTLs on 2A (*Xgwm328*), 2DL (*Xwmc41*), 3BS (*Xwmc754*), 5AL (*Xwmc415*), and 6DS (*Xcfd49*) (Liu *et al.* 2005b, Nishio *et al.* 2010, Suzuki *et al.* 2015, Yamashita *et al.* 2020, Zhu *et al.* 2012). The highest number of markers (28) were mapped to chromosome 2B and the lowest number (5) to chromosome 5D, with an average of 15.9 markers per chromosome. The results of MQM mapping demonstrated that a major QTL for WYMV resistance was detected on chromosome 2A in all environments (Fig. 3, Table 4). We designated the locus as *Q.Ymhk*. On the RefSeq of ‘Chinese Spring’ v2.1 (Zhu *et al.* 2021), the LOD peak of *Q.Ymhk* is located in the 5.9 Mb region between markers *tarc0497* and *snp4212* (or *snp4215*) on the short arm of chromosome 2A. The 151 RILs were classified into two groups that harbored either the ‘Hokkai 240’ or ‘Nanbukomugi’ genotypes at marker *snp4212*. Genotype of the ‘Hokkai 240’ showed number of uninfected lines than genotype of the ‘Nanbukomugi’ (Table 5). This result indicated that *snp4212* could be used for distinguishing the resistant genotype. Modern varieties were genotyped using *snp4212*, a flanking marker converted to a KASP marker. All of the four winter wheat varieties (‘Yumechikara’, ‘Kitahonami’, ‘Kitanokaori’, and ‘Tsurukichi’) in Hokkaido possessed genotype of ‘Nanbukomugi’.

## Discussion

Previously, Ohto *et al.* (2006) determined that ‘Hokkai 240’ was susceptible to Pathotype III because it could be infected with WYMV and showed distinct symptoms of the disease based on its response in an inoculation test. The results of this study were consistent with those from Ohto *et al.* (2006), in that infected individuals of the ‘Hokkai 240’ were noted as well in the ‘Nanbukomugi’ and ‘Fukuhokomugi’. Similarly, the number of infected plants obtained by testing infected leaves was clearly lower than those obtained from the other susceptible varieties in this study. We hypothesized that ‘Hokkai 240’ had the same resistance mechanism to Pathotype III as to the other pathotypes. In addition, our study demonstrated that ‘Hokkai 240’ was also resistant to Pathotype III in the field. These results suggest that ‘Hokkai 240’ had some function in inhibiting infection against all pathotypes in the field and in inoculation tests.

QTLs associated with Pathotype II and/or Pathotype III resistance have been identified on chromosomes 2DL, 3BS, and 6DS (Kobayashi *et al.* 2020, Nishio *et al.* 2010, Suzuki *et al.* 2015, Takeuchi *et al.* 2010, Yamashita *et al.* 2020). *Q.Ymhk* was detected on chromosome 2A, and contributed to resistance for Pathotype II and III (Fig. 3, Table 4). In a previous study, a gene named *YmNM* for WYMV resistance was mapped to chromosome 2A in a linkage analysis using AFLP and simple sequence repeat markers in the F_2_ populations of ‘Nigmai 9’ and ‘Yangmai 10’ (Liu *et al.* 2005b). With reference to the E2/M5 marker and *Xgwm328* that flank *YmNM*, the location of the former on the reference genome is unknown, while that of the latter is at 588 Mb (long arm range) in RefSeq v2.1 of ‘Chinse spring’ (Zhu *et al.* 2021). Therefore, *Q.Ymhk* is likely to be a novel QTL that is unique from *YmNM*.

‘Nanbukomugi’ possess a resistance allele at QTL on 5AL that confers partial resistance to Pathotype I (Kojima *et al.* 2019); therefore, no field test with Pathotype I was conducted. Pathotype III was isolated from infected plants of the variety ‘Shiroganekomugi’, which is resistant to Pathotype I. Pathotype III is considered to be a more infectious strain derived from Pathotype I (Ohki *et al.* 2014, Ohto *et al.* 2006). Because Pathotype III is considered a mutant strain of Pathotype I, *Q.Ymhk* is probably associated with resistance to Pathotype I as well. The hypothesis is also supported by the fact that ‘Hokkai 240’ is not infected with Pathotype I in inoculation test.

The analysis of genotypes of the marker (*snp4212*) tightly linked to *Q.Ymhk* suggested that the major wheat varieties in Hokkaido do not possess ‘Hokkai 240’ allele at the *Q.Ymhk*. Therefore, it would be beneficial to utilize ‘Hokkai 240’ allele in breeding programs. KASP marker is a robust tool for high-throughput and cost-effective selection (Rasheed *et al.* 2016). Genotyping with the KASP marker developed in this study demonstrated that all major varieties in Hokkaido possessed the genotype of ‘Nanbukomugi’. This marker will be useful for introducing the ‘Hokkai 240’ allele at the *Q.Ymhk* in breeding for WYMV resistance. Although, it should be noted that the resistance derived from the resistance allele of *Q.Ymhk* could be incomplete. Because previous studies have shown that ‘Hokkai 240’ was infected with WYMV at low frequencies in the field infested with Pathotype II (Yamashita *et al.* 2020). Therefore, it is necessary not only to introduce the resistance allele of *Q.Ymhk* singly but also to accumulate different resistance genes.

Mishina *et al.* (2023) evaluated differences in the quantity of WYMV depending on the combinations of alleles at the *Ym1* and *Ym2* loci with inoculation of leaves and exposed to virus-infested soil. The results suggested that *Ym1* was associated with resistance in leaves and roots while *Ym2* was associated with resistance in roots. ‘Yumechikara’ possesses the resistance allele at *Q.Ymym* located in similar genetic region to *Ym1* on 2DL (Kojima *et al.* 2015). ‘Yumechikara’ was resistant to the three WYMV pathotypes in field tests; however, this variety showed susceptibility to the disease following inoculation tests with the three pathotypes, suggesting that resistance of *Q.Ymym* expressed exclusively in the roots, in contrast to *Ym1*. The clear disease symptoms observed in ‘Yumechikara’ by inoculation tests might have been due to the allelic difference at the *Ym1* locus or the involvement of additional genes. Compared to ‘Yumechikara’, ‘Hokkai 240’ is an interesting genetic resource because it expresses broad resistance to WYMV in the above-ground parts (shoots). The causal gene for this resistance is likely to be *Q.Ymhk*, which could also be expressing resistance in the under-ground parts (roots). Although there are possibilities that resistance by *Q.Ymhk* could be related to viral suppression in shoot or inhibition of migration from root to shoot, these possibilities remain to be analyzed in detail.

*Q.Ymhk* is located in the region of a telomere, where the frequency of recombination is relatively higher than centromeric regions in wheat (Brazier and Glemin 2022, Osman *et al.* 2021). In addition, the phenotypes by *Q.Ymhk* could be clearly evaluated in inoculation test. Therefore, *Q.Ymhk* is considered to be an ideal target for cloning of the resistance gene. Understanding the resistance mechanism of this gene shall provide novel insights into WYMV resistance.

## Author Contribution Statement

KH developed experimental materials. KK, TO, and KH designed the experiments. KK, YT, SO, MF, MI, and KH performed the field experiments. KK, TO, and MS performed ELISA. TO performed inoculation tests. KK and GI performed genetic analysis. KK and KH analyzed the data and wrote the manuscript. All authors were involved in improving this manuscript.

## Supplementary Material

Supplemental Tables

## Figures and Tables

**Table 1.** Disease index (DI) of ‘Hokkai 240’ and ‘Nanbukomugi’ in the field test against Pathotype III

Variety and line	3-year mean DI	2005	2006	2007
Hokkai 240	0.3	0 (2)	0.3 (3)	0.7 (3)
Nanbukomugi	2.2	1.5 (2)	2.7 (3)	2.2 (5)

Numbers in parentheses are replications.

**Table 2.** Number of infected plants of three differential varieties and ‘Yumechikara’ with wheat yellow mosaic virus (WYMV) Pathotype I–III by mechanical inoculation

Varieties and line	Pathotype I		Pathotype II		Pathotype III	
	6-week	8-week	6-week	8-week	6-week	8-week
Hokkai 240	0/24*^a^*	0/24	0/23	0/23	0/24	8/24
Nanbukomugi	15/20	16/20	16/20	16/20	19/23	22/23
Fukuhokomugi	18/24	18/24	0/23	0/23	16/23	18/23
Yumechikara	10/24	NA*^b^*	7/23	NA	7/22	NA

*^a^* Number of infected plants/number of tested plants. *^b^* Not analyzed because most plants showed severe mosaic symptoms derived from WYMV.

**Table 3.** Number of infected plots for ‘Hokkai 240’ and ‘Nanbukomugi’ in the field test against Pathotype II

Year	Parents	
	Hokkai 240	Nanbukomugi
2017	0/8 (0%)*^a^*	3/6 (50%)
2018	0/6 (0%)	2/6 (33%)

*^a^* Infected plot frequency = number of infected plots/total number of plots studied. Numbers in parentheses are infection rate.

**Table 4.** Major quantitative trait loci on chromosome 2A for WYMV resistance detected in recombinant inbred lines (RILs) derived from the cross between ‘Nanbukomugi’ and ‘Hokkai 240’

Pathotype	Year	Trait	Position (cM)	Flanking marker	LOD*^a^*	LOD threshold	PVE (%)*^b^*	Additive effect*^c^*
III	2006	DI	15.7	*snp4212*, *snp4215*	15.5	3.9	38.4	0.83
	2007	DI	17.7	*snp4212*, *snp4215*	8.3	4.0	23.2	0.46
II	2017	ELISA	17.7	*snp4212*, *snp4215*	18.3	3.6	43.8	22.0
	2018	ELISA	16.7	*snp4212*, *snp4215*	10.4	3.9	27.1	15.8

*^a^* LOD = Logarithm of odds. *^b^* PVE = Percentage of variance explained. *^c^* Values indicate the effects of increasing trait value by the Nanbukomugi allele.

**Table 5.** Relationships between the resistance to Pathotype II and the genotype of flanking marker (*snp4212*) in RILs according to chi-square tests for independence

Genotype	2017			2018	
	Uninfected	Infected		Uninfected	Infected
Hokkai 240	66	4		57	13
Nanbukomugi	12	64		25	56
χ^2^	90.2			38.7	
*P*-value	2.12 × 10^–21^			4.97 × 10^–10^	

Chi-square test at 0.1% level. χ^2^_0.001_ = 10.8.
