# Supplementary material for: Identification of a major QTL conferring resistance to wheat yellow mosaic virus derived from the winter wheat ‘Hokkai 240’ on chromosome 2AS
Source: Breed Sci. 2024 Jul 18;74(3):232–9. doi: 10.1270/jsbbs.23079 (PMC11561412; doi:10.1270/jsbbs.23079)
Supplement: Supplementary file 1 — Supplemental Tables [file 74_232-s1.pdf]

doi: 10.1270/jsbbs.23079

Supplemental Table 1. Tested plant materials and their characterization to wheat yellow mosaic virus (WYMV) in this study

Varieties and line	Reaction to WYMV[1)]					
	Field			Inoculation		
	I	II	III	I	II	III
Hokkai 240	R[2)]	R[3)]	**U**	R[3)]	R[3)]	**S[3)*]**
Nanbukomugi	R[3)]	S[4)]	S[4)]	S[3)]	S[3)]	S[3)]
Fukuhokomugi	S[4)]	R[5)]	S[4)]	S[3)]	R[3)]	S[3)]
Yumechikara	R[6)]	R[7)]	R[8)]	U	U	U

1) "R", "S" and "U" indicate "resistant", "susceptible" and "unknown"
2) Oda and kashiwazaki (1989)
3) Ohto *et al*. (2006)
4) Kojima *et al*. (2019)
5) Yamashita *et al*. (2020)
6) Kojima *et al*. (2015)
7) Tabiki *et al*. (2011)
8) Kobayashi *et al*. (2020)
* The number of infected plants was lower than other varieties (Ohto *et al*. 2006)

Supplemental Table 2. Flanking markers of the previously reported resistance quantitative trait locus used in this study

Chromosome	Marker name	Forward sequence	Reverse sequence	Reference
2A	*Xgwm328*	GCAATCCACGAGAAGAGAGG	CACAAACTCTTGACATGTGCG	Somers *et al*. 2004, Liu *et al*. 2005
2DL	*SPT20446*	CTTAACACGAGAAGCTTGCA	TTATTCGAGGGAAAACTGGT	Kobayashi *et al*. 2020
2DL	*TNAC3149CD*	GCACCCTACTTCGGTACTGAC	TATTTGGGTTGTCATAATTCCTG	Kobayashi *et al*. 2020
2DL	*Xwmc41*	TCCCTCTTCCAAGCGCGGATAG	GGAGGAAGATCTCCCGGAGCAG	Somers *et al*. 2004, Nishio *et al*. 2010
2DL	*Xwmc181*	TCCTTGACCCCTTGCACTAACT	ATGGTTGGGAGCACTAGCTTGG	Somers *et al*. 2004, Nishio *et al*. 2010
3BS	*Xcfp59*	CGACACAAGGAGAGGCAAAG	AAATCAACCAGACCACCATTGC	Paux *et al*. 2008, Suzuki *et al*. 2015
3BS	*Xgpw7774*	GGCAACAACAACAACCAGG	CCATCCTTGCTCACATTCG	Paux *et al*. 2008 Suzuki. *et al*. 2015
3BS	*Xwmc754*	ATCCACATGAACCTCAACTTATGG	GGCATTGTTGTTGTACTGCAGTC	Somers *et al*. 2004, Suzuki *et al*. 2015
5AL	*Xwmc415*	AATTCGATACCTCTCACTCACG	TCAACTGCTACAACCTAGACCC	Somers *et al*. 2004, Zhu *et al*. 2012
6DS	*Xcfd49*	TGAGTTCTTCTGGTGAGGCA	GAATCGGTTCACAAGGGAAA	Somers *et al*. 2004, Yamashita *et al*. 2020

Supplemental Literature Cited

Kobayashi, F., H. Kojima, T. Tanaka, M. Saito, C. Kiribuchi-Otobe and T. Nakamura (2020) Characterization of the *Q.Ymym* region on wheat chromosome 2D associated with *wheat yellow mosaic virus* resistance. Plant Breed 139: 93-106.

Liu, W. H., H. Nie, Z. T. He, X. L. Chen, Y. P. Han, J. R. Wang, X. Li, C.G. Han and J. L. Yu (2005) Mapping of a wheat resistance gene to yellow mosaic disease by amplified fragment length polymorphism and simple sequence repeat markers. J Integr Plant Biol 47: 1133–1139.

Somers, D. J., P. Isaac and K. Edwards (2004) A high-density microsatellite consensus map for bread wheat (*Triticum aestivum* L.). Theor Appl Genet 109: 1105-1114.

Paux, E., P. Sourdille, J. Salse, C. Saintenac, F. Choulet, P. Leroy, A. Korol, M. Michalak, S. Kianian, W. Spielmeyer *et al.* (2008) A physical map of the 1-gigabase bread wheat chromosome 3B. Science 322: 101–104.

Zhu X., H. Wang, J. Guo, Z. Wu, A. Cao, T. Bie, M. Nie, F. M. You, Z. Cheng, J. Xiao *et al*. (2012) Mapping and validation of quantitative trait loci associated with wheat yellow mosaic bymovirus resistance in bread wheat. Theor Appl Genet 124: 177-188.

Supplemental Table 3. Disease index (DI) in recombinant inbred lines (RILs) over 2 years in field tests of Pathotype III

No.	RIL	2006	2007
		DI	DI
1	NHRILs-001	2	2
2	NHRILs-002	2	2
3	NHRILs-003	0	0
4	NHRILs-004	0	0
5	NHRILs-005	2	NA
6	NHRILs-006	NA*	0
7	NHRILs-007	2	0
8	NHRILs-008	4	3
9	NHRILs-009	3	2
10	NHRILs-010	0	2
11	NHRILs-011	4	2
12	NHRILs-012	2	2
13	NHRILs-013	2	1
14	NHRILs-014	1	1
15	NHRILs-015	4	1
16	NHRILs-016	1	2
17	NHRILs-017	2	2
18	NHRILs-018	1	1
19	NHRILs-019	0	1
20	NHRILs-020	0	0
21	NHRILs-021	3	2
22	NHRILs-023	2	0
23	NHRILs-024	0	0
24	NHRILs-025	1	0
25	NHRILs-027	2	2
26	NHRILs-028	2	1
27	NHRILs-029	2	1
28	NHRILs-030	1	0
29	NHRILs-031	2	2
30	NHRILs-032	2	2
31	NHRILs-033	2	2
32	NHRILs-034	2	2
33	NHRILs-035	2	0
34	NHRILs-036	3	2
35	NHRILs-037	3	2

36	NHRILs-038	0	1
37	NHRILs-039	2	1
38	NHRILs-040	1	0
39	NHRILs-041	3	2
40	NHRILs-042	0	2
41	NHRILs-043	3	2
42	NHRILs-044	2	2
43	NHRILs-045	0	0
44	NHRILs-046	1	2
45	NHRILs-047	2	0
46	NHRILs-048	0	0
47	NHRILs-049	3	2
48	NHRILs-050	0	0
49	NHRILs-051	0	0
50	NHRILs-052	0	0
51	NHRILs-053	0	1
52	NHRILs-054	3	3
53	NHRILs-055	1	0
54	NHRILs-056	3	2
55	NHRILs-057	3	2
56	NHRILs-058	3	2
57	NHRILs-059	1	NA
58	NHRILs-060	1	0
59	NHRILs-061	3	2
60	NHRILs-062	0	0
61	NHRILs-063	3	2
62	NHRILs-064	3	2
63	NHRILs-065	2	2
64	NHRILs-066	2	2
65	NHRILs-067	2	1
66	NHRILs-068	2	3
67	NHRILs-069	3	2
68	NHRILs-070	0	0
69	NHRILs-071	3	2
70	NHRILs-072	0	0
71	NHRILs-073	2	0
72	NHRILs-074	1	0
73	NHRILs-075	2	2
74	NHRILs-076	2	1

75	NHRILs-077	3	1
76	NHRILs-078	3	2
77	NHRILs-079	2	0
78	NHRILs-080	2	1
79	NHRILs-081	1	0
80	NHRILs-082	2	0
81	NHRILs-083	2	0
82	NHRILs-084	2	1
83	NHRILs-085	2	2
84	NHRILs-086	0	0
85	NHRILs-087	3	2
86	NHRILs-088	0	1
87	NHRILs-089	2	0
88	NHRILs-090	2	0
89	NHRILs-091	3	2
90	NHRILs-092	3	2
91	NHRILs-093	3	2
92	NHRILs-094	2	3
93	NHRILs-095	3	2
94	NHRILs-096	0	0
95	NHRILs-097	0	2
96	NHRILs-098	2	3
97	NHRILs-099	0	0
98	NHRILs-100	0	0
99	NHRILs-101	4	3
100	NHRILs-102	0	2
101	NHRILs-103	4	2
102	NHRILs-104	0	1
103	NHRILs-105	3	2
104	NHRILs-106	4	2
105	NHRILs-107	3	2
106	NHRILs-108	0	2
107	NHRILs-109	3	2
108	NHRILs-110	2	2
109	NHRILs-111	3	0
110	NHRILs-112	3	0
111	NHRILs-113	3	1
112	NHRILs-114	0	2
113	NHRILs-115	0	NA

114	NHRILs-116	0	0
115	NHRILs-117	3	0
116	NHRILs-118	4	2
117	NHRILs-119	NA	1
118	NHRILs-120	2	0
119	NHRILs-121	2	1
120	NHRILs-122	3	2
121	NHRILs-123	3	2
122	NHRILs-124	0	NA
123	NHRILs-125	3	NA
124	NHRILs-126	3	2
125	NHRILs-128	0	1
126	NHRILs-130	2	1
127	NHRILs-131	4	2
128	NHRILs-132	2	1
129	NHRILs-133	2	2
130	NHRILs-134	4	3
131	NHRILs-135	NA	2
132	NHRILs-136	0	1
133	NHRILs-137	1	2
134	NHRILs-138	0	0
135	NHRILs-139	2	0
136	NHRILs-140	3	2
137	NHRILs-141	2	2
138	NHRILs-142	3	1
139	NHRILs-143	3	2
140	NHRILs-144	2	2
141	NHRILs-145	4	2
142	NHRILs-146	2	0
143	NHRILs-147	0	0
144	NHRILs-148	0	2
145	NHRILs-149	NA	NA
146	NHRILs-150	0	1
147	NHRILs-151	1	1
148	NHRILs-152	0	2
149	NHRILs-153	1	1
150	NHRILs-154	1	1
151	NHRILs-155	4	2

* Not analyzed

Supplemental Table 4. Classification of resistance against Pathotype II evaluated by field test in RILs

2017	2018	No. of lines
Resistant	Resistant	58
Resistant	Susceptible	20
Susceptible	Resistant	21
Susceptible	Susceptible	47

The five lines were excluded, which could not be evaluated in 2017 due to snow or cold damage
